# The association between cardiac drug therapy and anxiety among cardiac patients: results from the national DenHeart survey

**DOI:** 10.1186/s12872-022-02724-4

**Published:** 2022-06-20

**Authors:** Camilla Rotvig, Anne Vinggaard Christensen, Knud Juel, Jesper Hastrup Svendsen, Martin Balslev Jørgensen, Trine Bernholdt Rasmussen, Britt Borregaard, Lars Thrysoee, Charlotte Brun Thorup, Rikke Elmose Mols, Selina Kikkenborg Berg

**Affiliations:** 1 The Heart Centre, Rigshospitalet, Copenhagen University Hospital, Copenhagen, Denmark; 2 National Institute of Public Health, University of Southern Denmark, Copenhagen, Denmark; 3 Department of Clinical Medicine, Faculty of Health and Medical Sciences, University of Copenhagen, Copenhagen, Denmark; 4 Psychiatric Centre Copenhagen, and Institute of Clinical Medicine, University of Copenhagen, Copenhagen, Denmark; 5 Department of Cardiology, Herlev and Gentofte University Hospital, Hellerup, Denmark; 6 Department of Cardiothoracic and Vascular Surgery, Odense University Hospital, Odense, Denmark; 7 Department of Cardiology, Odense University Hospital, Odense, Denmark; 8 Department of Cardiology and Department of Cardiothoracic Surgery and Clinical Nursing Research Unit, Aalborg University Hospital, Aalborg, Denmark; 9 Department of Cardiology, Aarhus University Hospital, Aarhus, Denmark

**Keywords:** Anxiety, Drug therapy, Drug-related side effects and adverse reactions, Health surveys, Heart diseases

## Abstract

**Background:**

Neuropsychiatric side effects of cardiac drugs such as nervousness, mood swings and agitation may be misinterpreted as symptoms of anxiety. Anxiety in cardiac patients is highly prevalent and associated with poor outcomes, thus an accurate identification is essential. The objectives were to: (I) describe the possible neuropsychiatric side effects of common cardiac drug therapies, (II) describe the use of cardiac drug therapy in cardiac patients with self-reported symptoms of anxiety compared to those with no symptoms of anxiety, and (III) investigate the association between the use of cardiac drug therapy and self-reported symptoms of anxiety.

**Methods:**

DenHeart is a large national cross-sectional survey combined with national register data. Symptoms of anxiety were measured by the Hospital Anxiety and Depression Scale (HADS-A) on patients with ischemic heart disease, arrhythmia, heart failure and heart valve disease. Side effects were obtained from ‘product summaries’, and data on redeemed prescriptions obtained from the Danish National Prescription Registry. Multivariate logistic regression analyses explored the association between cardiac drug therapies and symptoms of anxiety (HADS-A ≥ 8).

**Results:**

Among 8998 respondents 2891 (32%) reported symptoms of anxiety (HADS-A ≥ 8). Neuropsychiatric side effects were reported from digoxin, antiarrhythmics, beta-blockers, ACE-inhibitors and angiotensin receptor antagonists. Statistically significant higher odds of reporting HADS ≥ 8 was found in users of diuretics, lipid-lowering agents, nitrates, antiarrhythmics and beta-blockers compared to patients with no prescription.

**Conclusion:**

Some cardiac drugs were associated with self-reported symptoms of anxiety among patients with cardiac disease. Of these drugs neuropsychiatric side effects were only reported for antiarrhythmics and beta-blockers. Increased awareness about the possible adverse effects from these drugs are important.

**Supplementary Information:**

The online version contains supplementary material available at 10.1186/s12872-022-02724-4.

## Introduction

Anxiety is considered an established psychosocial risk factor within cardiovascular disease [1]. In Scandinavian countries, it has been documented that 20–25% of patients with cardiac diseases experience symptoms of anxiety, measured by the Hospital Anxiety and Depression Scale (HADS-A, anxiety) [2, 3]. Anxiety has been associated with poor health outcomes such as an increased risk of mortality, and cardiac events [4]. Thus, accurate identification of anxiety symptoms is essential to properly intervene in order to reduce its symptoms.

HADS-A is frequently used in clinical settings within cardiology to assess the patient’s level of anxiety [5]. It is well suited to explore anxiety symptoms in cardiac patients as it leaves out all physical symptoms of anxiety that may also be related to the underlying cardiac disease [5, 6]. Still, it is merely a screening tool, and a diagnostic interview is required to confirm an accurate diagnosis of an anxiety disorder [7]. In a randomised controlled trial of patients with an Implantable Cardioverter-Defibrillator (ICD), 76% of the patients that screened positive for symptoms of anxiety by self-report (HADS-A ≥ 8) also met the diagnostic criteria for an anxiety disorder assessed by the diagnostic instrument, the Structured Clinical Interview for DSM-IV (SCID) [8]. However, as 24% of patients did not fulfil the diagnostic criteria for an anxiety disorder by SCID although reporting a high level of anxiety symptoms, it appears to be necessary to investigate the phenomenon more thoroughly.

When measuring anxiety using HADS-A, tension, worry, fear, panic, difficulty in relaxing, and restlessness are included as symptoms indicating anxiety [5]. It has, however, been debated whether some of these symptoms may in fact be due to cardiac drug therapy, which has been known to cause or exacerbate symptoms [9, 10]. Patients with cardiac diseases are often prescribed beta-blockers, lipid-lowering agents, angiotensin-converting enzyme (ACE)-inhibitors, diuretics and antiarrhythmics to treat their underlying cardiac diseases. Despite the well-established benefits of these therapies, such as improving survival in patients with heart failure [11], myocardial infarction and arrhythmias [12], there is an ongoing debate on their possible consequences [10]. For example, the process of lipophilic beta-blockers (e.g. metoprolol) crossing the blood–brain barrier is thought to be associated with impaired psychological functioning [13]. Furthermore, the antiarrhythmic drug amiodarone has been linked with thyroid abnormalities in 15% of patients, and this can lead to mood, cognitive, and psychotic symptoms [14]. The existing original studies investigating cardiac drug therapies and the risk of developing symptoms of anxiety are conflicting and based on small sample sizes [15–17]. A newly published meta-analysis concluded that the use of cardiac drugs should be considered when evaluating depression/anxiety in patients with cardiovascular disease [9]. Yet, they identified a lack of original studies that focused on cardiovascular patients and studies including more than one type of cardiac drugs to investigate this topic [9]. Therefore, among patients with a cardiac diagnosis, the objectives of the current study were to: (I) describe the possible neuropsychiatric side effects to common cardiac drug therapies, (II) describe the use of cardiac drug therapy in patients with self-reported symptoms of anxiety compared to those with no symptoms of anxiety, and (III) investigate the association between the use of cardiac drug therapy and self-reported symptoms of anxiety.

## Methods

### Design

The current study is based on data from the national survey DenHeart combined with Danish national registers. The study methods of DenHeart are previously published [18], and a summary will be presented in the following.

### Patient population

In the DenHeart study, all patients with a cardiac disease discharged from one of five Danish heart centres were included from April 2013 to April 2014. Patients > 18 years were asked to fill out a questionnaire at hospital discharge or within three days post-discharge including validated instruments, as well as HADS-A. Patients were included in the current study if they: (a) were diagnosed with ischemic heart disease, arrhythmia, heart failure or valvular heart disease prior to hospitalisation and (b) had completed HADS-A questionnaire.

### Data from national registers

To link DenHeart survey data with national registries, a unique personal identification number was used, obtained from the Danish Civil Registration System (CRS) [19]. From CRS, data on sex, age and marital status were derived. Educational levels were obtained from the Danish Education Register [20] and were divided into primary school (≤ 10 years), upper secondary/vocational education and higher education.

Information on co-morbidity and cardiac diagnoses was obtained from the Danish National Patient Register [21]. To calculate the Tu-comorbidity index score and the Charlson comorbidity index, co-morbidities were linked as a secondary diagnosis going 10 years back, not including the index admission. The Tu-comorbidity index score and Charlson comorbidity index, is a composite score of different co-morbidities, and each co-morbidity accounts for one point, and zero equals no co-morbidity [22]. Diagnoses included were based on the following ICD-10 codes; ischemic heart disease: I20-I25, T82.3D, Z95.1, Z95.5, arrhythmia: I44-I45, I47-I49, Z95.0, I46.0, I46.9, R00.0, R00.1, R00.2, R00.8A, T75.0, T75.4, T82.1, T82.8, heart failure: I50, I42.0-I43.8, I11.0, I51.7, R57.0 and heart valve disease: I05.0-I06.0, I34.0-I37.2, Z95.2-Z95.4, I39.1, I39.2, I51.1A. Information on psychotropic medication (Selective Serotonin Reuptake Inhibitors, Tricyclic Antidepressants, Serotonin–norepinephrine reuptake inhibitor, benzodiazepines, melatonin, first generation antipsychotics and second generation antipsychotics) was identified from the Danish National Prescription Registry (DNPR) and to be defined as a user, at least one dispensed prescription within one year prior to completion of HADS-A was required [23].

### Neuropsychiatric side effects

A side effect is an unwanted effect of a drug. In every European country, the safety of medicines is monitored by a medicines agency that exchanges reports with The European Medicines Agency. Once a drug is approved, reporting of suspected side effects is important and enables continuous monitoring of the benefits versus risks of a drug [24]. Side effects can be retrieved from a ‘product summary’ that follows every approved drug and is updated once a week [25, 26]. The product summary includes all adverse reactions from clinical trials, safety studies, epidemiological studies and/or evaluation of causality from individual case reports. Furthermore, after approval of the drug, reports from health care professionals or the individual who experienced a side effect are also included [27]. The frequency of side effects is defined in the following conventions: ‘very common’ (1–10%), ‘common’ (0,1–1%) or ‘rare’ (0,01–0,1%) [28]. For the present study, neuropsychiatric side effects were defined as those reported in the ‘product summary’ of the 11 included types of cardiac drugs. Furthermore, for the current study, side effects had to be comparable to one of the symptoms included in HADS-A; tension, worry, fear, panic, difficulty in relaxing, and restlessness[5]. Side effects were presented in a table divided by frequency and type of drug. If the same side effect was represented for the same type of drug more than once, the most frequent was included.

### Cardiac drug therapy

In the current study, cardiac drugs were defined as drugs commonly used when treating ischemic heart disease, arrhythmia, heart failure and valvular heart disease [29], and included the following 11 types: Anticoagulants (B01A): Phenprocoumon, Warfarin, Pradaxa, Eliquis, Xarelto and Lixiana. Aspirins (B01AC06): Acetylsalicylic acid. Digoxin (C01AA): Digoxin. Antiarrhythmics (C01B): Amiodaron, Dronedaron, Flecainid, Verapamil, Propafenon and Sotalol. Nitrates (C01DA): Isorbide dinitrate. Diuretics (C03): Hydrochlorothiazide, Metolazone, Bumetanide, Furosemide, Spironolactone and Eplerenone. Aldosterone antagonists (C03DA): Spironolactone and Eplerenone. Beta-blockers (C07): Atenolol, Bisoprolol, Metoprolol, Esmolol, Nebivolol, Landiolol, Propranolol and Sotalol. ACE-inhibitors (C09AA): Lisinopril, Captopril, Enalapril, Perindopril, Trandolapril and Ramipril. Angiotensin receptor antagonists (C09CA): Losartan, Irbesartan, Candesartancilexetil, Valsartan, Telmisartan and Olmesartanmedoxomil. Lipid-lowering Agents (C10AA): Simvastatin, Pravastatin, Fluvastatin, Atorvastatin and Rosuvastatin (Additional file 1: Table S1). Redeemed prescriptions of cardiac drug therapies were identified from DNPR [23]. The DNPR was established in 1994 and contains information on all dispensed prescriptions in Denmark. The Anatomical Therapeutic Chemical Classification code (ATC) and the date of dispensing were obtained from DNPR. To be defined as a user of cardiac drugs, at least one dispensed prescription within six months prior to completion of HADS-A was required.

### Strengths and limitations

The large sample size, the national multicenter design and inclusion of patients with a known cardiac disease were the major strengths of this study and increased generalisability. However, the cross-sectional design prevented us from evaluating the causal relationship between the use of cardiac drugs and symptoms of anxiety. Furthermore, reverse causality is possible hence the results must be interpreted with caution. Moreover, only a minor proportion of the actual side effects is reported, in general. Thus, it has been found that < 1% of all adverse reactions leading to hospitalisation are reported [30]. Therefore, the actual presence of neuropsychiatric side effects may be underestimated. Notably, we did not consider the dosage of cardiac drugs and that the pharmacological effects within each ATC group may have different effects, which may play an essential role in the association and furthermore we cannot exclude drug-drug interaction. Although we can only account for prescribed drugs, we cannot exclude that lack of drug adherence may have contributed to misclassification in users. Symptoms of anxiety were defined by the screening tool HADS-A. Although HADS-A might overestimate the prevalence of anxiety, HADS-A ≥ 8 is known to be a valid and reliable indicator of anxiety within this population [1, 31]. Among patients with HADS-A < 8 versus HADS-A ≥ 8 of marital status, lifestyle factors, educational level and co-morbidities differed. The behavioural pathways of anxiety are linked to risks in health behaviour, such as smoking, overeating, consuming drugs/alcohol, physical inactivity, lower educational level and living alone [32]. However, it is not possible to control for all potential confounders in register-based research and thus residual confounding is likely.

### Patient-reported data

HADS is a validated 14-item questionnaire that assesses the level of symptoms of both anxiety (HADS-A) and depression (HADS-D) [5]. The two sub-scales, HADS-A and HADS-D, includes seven questions each. HADS has been validated in a Danish population of patients with a cardiac diagnosis, with a Cronbach’s alpha of 0,87 for HADS-A and 0,82 for HADS-D.[31] In the current study, only data from the HADS-A was used.

The items included in HADS-A focus on general anxiety with five items being in line with the diagnostic criteria for general anxiety. For the present study, a score of eight or above on HADS-A suggested the presence of a mood disorder, as recommended by the developers [5].

Self-reported data on lifestyle factors including information on height and weight to calculate body mass index (BMI) (kg)/height^2^ (m^2^), smoking behaviour and alcohol intake were included from the DenHeart Survey.

### Statistical methods

Demographic data were presented as the proportion with numbers and percentages (%) for categorical variables and as means and standard deviations (SD) for continuous measures.

To describe the distribution of cardiac drug therapies divided by HADS-A < 8 and HADS-A ≥ 8, the proportion with numbers, percentages (%), and probability values (p-value) were used.

Multivariate logistic regression analyses were used to explore the association between the use of cardiac drugs and the presence of symptoms of anxiety (HADS-A ≥ 8). The results were presented as odds ratios (OR) with 95% confidence intervals (CI) for unadjusted and adjusted analyses. The logistic regression analyses were performed as three models: Model 1, the crude model; Model 2, adjusted for age, sex, diagnosis group and TU-comorbidity index score; and Model 3, adjusted for age, sex, diagnosis group, TU-comorbidity index score, cardiac drug group and psychotropic medication.

Statistical analyses were performed using SAS V.9.4.

## Results

### Patient characteristics

**Fig. 1:**
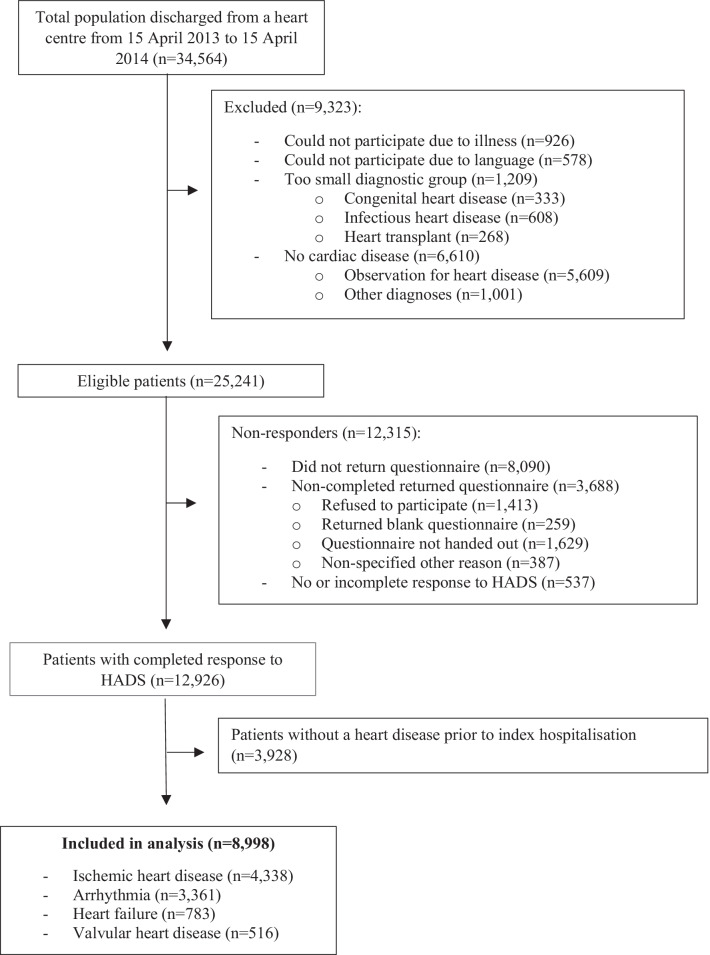
Flow chart

HADS-A section was completed by 8998 patients with an already known cardiac disease: ischemic heart disease, arrhythmia, heart failure or valvular heart disease failure (Fig. 1). Of these, 2891 (32%) reported HADS-A ≥ 8. The mean age was 67 years (SD 12) for patients with HADS-A < 8, and 64 years (SD 12) for HADS-A ≥ 8. Among patients with HADS-A < 8 vs. HADS-A ≥ 8, notable differences in demographic characteristics included: Smoking daily (10% vs. 15%), being divorced (12% vs. 17%), BMI ≥ 30 (24% vs. 28%), high alcohol intake (7% vs. 8%), basic educational level (30% vs. 35%), and ≥ 3 co-morbidities (11% vs. 14%) (Table 1).

**Table 1:** Demographic and clinical profile for patients with cardiac disease stratified by HADS-A (n = 8998)

	HADS-A < 8 n = 6107 (68)	HADS-A ≥ 8 n = 2891 (32)	*p values*
	n (%)	n (%)	
Sex			< 0.0001^**a**^
Male	4491 (74)	1841 (64)	
Age, mean (SD)	67 (12)	64 (12)	< 0.0001^**b**^
Diagnosis	0.0003		
Ischemic heart disease	2850 (47)	1488 (51)	
Arrhythmia	2363 (39)	998 (35)	
Heart failure	540 (9)	243 (8)	
Valvular heart disease	354 (6)	162 (6)	
Length of stay, mean (SD)	2.1 (2.8)	2.4 (3.3)	0.0002^**c**^
Co-morbidity			–
Hypertension	2524 (41)	1363 (47)	
Diabetes	823 (13)	538 (19)	
Ventricular arrhythmia	396 (6)	199 (7)	
Ischaemic heart disease	3700 (61)	1889 (65)	
Myocardial infarction	1596 (26)	834 (29)	
PCI	1547 (25)	787 (27)	
CABG	374 (6)	187 (6)	
Heart failure	1481 (24)	759 (26)	
Renal disease	225 (4)	128 (4)	
Chronic obstructive pulmonary disease	529 (9)	381 (13)	
Smoking (daily use) ^d^	< 0.0001		
Yes		621 (10)	420 (15)
BMI ≥ 30 ^d^	0.0005		
Yes		1392 (24)	746 (28)
Alcohol > recommendation ^d, e^	0.1850		
Yes		397 (7)	206 (8)
Marital status	< 0.0001		
Married		4050 (66)	1751 (61)
Divorced	751 (12)	480 (17)	
Widowed	778 (13)	358 (12)	
Unmarried	528 (7)	302 (10)	
Educational level ^d^			< 0.0001
Higher	1517 (25)	539 (19)	
Upper secondary or vocational	2660 (44)	1285 (46)	
Basic	1820 (30)	988 (35)	
Tu-comorbidity index score	0.0081		
0	1407 (23)	640 (22)	
1	2596 (43)	1220 (42)	
2	1403 (23)	628 (22)	
3 +	701 (11)	403 (14)	
Charlson comorbidity index			< 0.0001
0	2118 (35)	887 (31)	
1	1795 (29)	816 (28)	
2	1061 (17)	493 (17)	
3 +	1133 (19)	695 (24)	
Psychotropic medication			0.1442
Yes	119 (1.9)	70 (2.4)	

^**a**^χ^2^ test

^**b**^T-test

^**c**^Non-parametric Wilcoxon signed rank test

^**d**^Missing data from 1 to 8%

^**e**^Alcohol intake above high-risk limit is defined by The Danish National Board of Health to be a weekly intake > 21 standard drinks for men and > 14 standard drinks for women

### Neuropsychiatric side effects

Neuropsychiatric side effects identified from ‘product summaries’ [33] within the included cardiac drug therapies were: irritability, anxiety, mood swings, nervousness, restlessness, mental disorder and agitation (Table 2). Within five of the 11 included drugs, neuropsychiatric side effects were reported as either “very common”, “common” or “rare”. In six types of cardiac drugs, no neuropsychiatric side effects comparable to the symptoms in HADS-A were reported. In beta-blockers, anxiety and nervousness were reported as very common. In antiarrhythmics, the following side effects were very common: anxiety, mood swings and nervousness. Across the five cardiac drugs nervousness, anxiety and mood swings were the three most reported side effects. Within anticoagulants, aspirins, nitrates, diuretics, aldosterone antagonists and lipid-lowering agents, no neuropsychiatric side effects as defined in the current study were identified from the ‘product summaries’ (Table 2).

**Table 2:** N side effects from ‘product summaries’ of each group of cardiac drugs

	Very common (1%–10%)	Common (0.1%–1%)	Rare (0.01%–0.1%)
Diuretics			
Lipid-lowering agents			
Aspirins			
Anticoagulants			
Aldosterone antagonists			
Nitrates			
Antiarrhythmics	Anxiety Mood swings Nervousness		
Digoxin		Irritability	
Angiotensin receptor antagonists			Anxiety
ACE-inhibitors		Mood swings Nervousness Anxiety Restlessness	Mental disorder Agitation
Beta-blockers	Anxiety Nervousness	Restlessness	Mood swings

### The use of cardiac drug therapies

A statistically significant difference was found between cardiac drug users reporting HADS-A < 8 and HADS-A ≥ 8 within diuretics (52% vs. 55%, p = 0.012), lipid lowering agents (73% vs. 76%, p = 0.002), aspirins (70% vs. 73%, p = 0.032), nitrates (37% vs. 43%, p < 0.001), antiarrhythmics (16% vs. 18%, p = 0.036), and beta-blockers (81% vs. 83%, p = 0.010) (Table 3).

**Table 3:** Descriptive statistics showing the distribution of cardiac drug therapies and HADS-A

	HADS-A < 8 n = 6107 n (%)	HADS-A ≥ 8 n = 2891 n (%)	*p values*
Diuretics	3162 (52)	1579 (55)	0.012
Lipid-lowering Agents	4449 (73)	2194 (76)	0.002
Aspirins	4304 (70)	2101 (73)	0.032
Anticoagulants	5681 (93)	2676 (93)	0.43
Aldosterone antagonists	1158 (19)	566 (20)	0.48
Nitrates	2234 (37)	1234 (43)	< 0.001
Antiarrhythmics	978 (16)	514 (18)	0.036
Digoxin	841 (14)	416 (14)	0.43
Angiotensin receptor antagonists	1436 (24)	712 (25)	0.25
ACE-inhibitors	2548 (42)	1200 (42)	0.85
Beta-blockers	4921 (81)	2395 (83)	0.010

### The association between the use of cardiac drug therapies and symptoms of anxiety

In Table 4, results of the adjusted Model 3 revealed that redeeming a prescription within five types of cardiac drugs were associated with statistically significant higher odds of reporting HADS ≥ 8, compared to non-users; diuretics (OR: 1.14, 95% CI: 1.02;1.28) lipid-lowering agents (OR: 1.19, 95% CI: 1.05;1.36), nitrates (OR: 1.26, 95% CI: 1.13;1.40), antiarrhythmics (OR: 1.25, 95% CI: 1.10;1.43), and beta-blockers (OR: 1.14, 95% CI: 1.01;1.29).

**Table 4:** The association between the use of cardiac drug therapies and symptoms of anxiety

	Model 1^a^	Model 2^b^	Model 3^c^
	OR (95% CI)	OR (95% CI)	OR (95% CI)
*HADS-A ≥ 8 n = 2891*			
Diuretics	1.12 (1.03; 1.23)*	1.18 (1.07; 1.31)*	1.14 (1.02; 1.28)*
Lipid-lowering agents	1.17 (1.06; 1.30)*	1.27 (1.12; 1.44)*	1.19 (1.05; 1.36)*
Aspirin	1.11 (1.01; 1.23)*	1.16 (1.03; 1.30)*	1.10 (0.96; 1.25)
Anticoagulants	0.93 (0.79; 1.11)	1.19 (0.99; 1.44)	0.93 (0.75; 1.14)
Aldosterone antagonists	1.04 (0.93; 1.16)	1.02 (0.90; 1.16)	0.91 (0.79; 1.04)
Nitrates	1.29 (1.18; 1.41)*	1.30 (1.17; 1.44)*	1.26 (1.13; 1.40)*
Antiarrhythmics	1.13 (1.01; 1.28)*	1.27 (1.12; 1.44)*	1.25 (1.10; 1.43)*
Digoxin	1.05 (0.93; 1.20)	1.13 (0.98; 1.29)	1.10 (0.95; 1.26)
Angiotensin receptor antagonists	1.06 (0.96; 1.18)	1.08 (0.97; 1.20)	1.04 (0.98; 1.16)
ACE-inhibitors	0.99 (0.91; 1.08)	1.02 (0.93; 1.12)	0.98 (0.88; 1.08)
Beta-blockers	1.16 (1.04; 1.31)*	1.23 (1.09; 1.39)*	1.14 (1.01; 1.29)*

*Statistically significant association

^a^Multivariable logistic regression model unadjusted

^b^Multivariable logistic regression model adjusted for age, sex, TU-comorbidity index score and diagnostic group

^c^Multivariable logistic regression model adjusted for age, sex, TU-comorbidity index score, diagnostic group, cardiac drug group and psychotropic medication

## Discussion

The results of this cross-sectional study revealed that neuropsychiatric side effects were reported for digoxin, antiarrhythmics, beta-blockers, ACE-inhibitors and angiotensin receptor antagonists. In a population including patients with ischemic heart disease, arrhythmia, heart failure or valvular heart disease, the use of six different types of cardiac drugs (diuretics, lipid-lowering agents, aspirins, nitrates, antiarrhythmics, and beta-blockers) was significantly more common in patients reporting symptoms of anxiety vs. no symptoms of anxiety. In adjusted analysis, an association between five different types of cardiac drug therapies (diuretics, lipid-lowering agents, nitrates, antiarrhythmics, and beta-blockers) and symptoms of anxiety was demonstrated.

For five types of cardiac drugs neuropsychiatric side effects in relation to anxiety were reported. This included antiarrhythmics, digoxin, angiotensin receptor antagonists, ACE-inhibitors and beta-blockers. However, of these only users of antiarrhythmics and beta-blockers were statistically associated with higher odds of reporting symptoms of anxiety that could be related to HADS-A. Diuretics, lipid-lowering agents and nitrates were associated with higher odds of reporting symptoms of anxiety likewise, but side effects similar to anxiety were not reported in ‘product summaries’. Therefore, a direct causative link between the use of cardiac drug therapy and symptoms of anxiety is difficult to establish.

For the current study a 14% higher odds of anxiety was found in patients prescribed with diuretics. A recent meta-analysis revealed evidence that supports this association. By pooling data from 10.391 participants with coronary artery disease the study found a 39% higher odds of anxiety (OR:1.39, 95% CI:1.26;1.52) for users of diuretics [34]. Diuretics are highly relevant in the treatment of heart failure and hypertension as the drug helps reducing the fluid load, but on the other hand it can drain the sodium of the body and hereby affect the autonomic nervous system [35]. Long-term use may therefore contribute to damaging the autonomic nervous system by electrolytes disturbances and indirectly affect an anxiolytic response.

The absence of an association within lipid-lowering agents and symptoms of anxiety in a study of patients with ischemic heart disease (n = 606) is conflicting with the results of the current study. We found a 19% higher odds of reporting symptoms of anxiety in users of lipid-lowering agents. A meta-analysis including studies of patients with cardiovascular disease found no such association either [34]. However, compared to controls a study proposes that high doses of statins are associated with major depressive disorder in an Austrian population (n = 7,481,168) [36]. Therefore, the dose of statins may be taken into consideration when prescribing.

In the current study the use of nitrates was associated with a 26% higher odds of reporting symptoms of anxiety. No studies in humans has been identified investigating this link, but the use of nitrates has earlier been associated with a 32% higher odds of depression in patients with cardiovascular disease [9]. However, to our knowledge no research explains the mechanisms that links nitrates with depression or anxiety why more research into this field is needed.

In the current study, antiarrhythmic therapy was associated with 25% higher odds of reporting symptoms of anxiety. Antiarrhythmics are used in the treatment of atrial fibrillation, atrial flutter, ventricular arrhythmias and ventricular fibrillation. The use of antiarrhythmics has been associated with thyroid abnormalities, which can lead to dysregulation that causes mood, cognitive and psychotic symptoms [10]. Yet, anxiety symptoms measured by HADS-A were not associated with the use of antiarrhythmics in a cohort study of 378 patients with atrial fibrillation [15].

A 14% increased likelihood of symptoms of anxiety among users of beta-blockers was demonstrated. Beta-blockers inhibit β-adrenergic receptors and are used in the treatment of hypertension, heart failure, ischemic heart disease and arrhythmias [10]. The process of lipophilic beta-blockers that passes the blood–brain barrier may partly explain this association. It is thought to affect the synthesis and reuptake of neurotransmitters and thereby impact psychological functioning [13]. Furthermore, a study propose that β_1_-adrenoceptors play an important role in the basolateral amygdalae when looking at anxiety-like behaviour [13]. This suggests that beta-blocker therapy may produce an anxiolytic response. By contrast, a study of patients with ICD (n = 448) found no association between beta-blockers and symptoms of anxiety [17]. Moreover, a meta-analysis did not find any association between beta-blocker use and anxiety for patients with cardiovascular disease [9].

The underlying mechanisms of the association between cardiac drugs and developing symptoms of anxiety are not well understood. Several factors may complicate whether cardiac drugs cause a given anxiety symptom, and a number of other explanations need to be considered when interpreting the results. First, neuropsychiatric symptoms are common among cardiac patients [10], which is reflected in the high prevalence of anxiety in patients with cardiac disease [2, 3]. Next, the symptoms of anxiety may be a natural adjustment response to the onset of a cardiac disease and might be unrelated to an anxiety disorder or the side effects of cardiac drug therapy [10]. Third, the known proclivity of individuals who have psychological diagnoses to develop cardiovascular disease is well reported [6, 37]. Hence, it is possible that some patients had anxiety prior to the onset of the cardiovascular disease and the symptoms may not even be related to cardiac drug therapy. However, after adjusting for psychotropic medication that was used as a proxy for mental health disorders associations remained.

Since anxiety is a common problem in patients with a cardiac disease, and both decreased mental and physical health is likely [2], the drug therapy should be individualised and strive for treatment of both psychological and physical problems. The benefits of cardiac drug therapies exceed the disadvantages in most cases. However, the risk-benefits of a drug should always be considered, including risks such as side effects, dose and duration. This study adds knowledge to the associations on anxiety symptoms as side effects of cardiac drugs, but further research is needed into the link between cardiac drug therapy and anxiety to make fully informed prescriptions for cardiac patients.

## Conclusion

Certain cardiac drugs may cause symptoms of anxiety as a side effect. In patients with ischemic heart disease, arrhythmia, heart failure and valvular heart disease diuretics, lipid-lowering agents, nitrates, antiarrhythmics, and beta-blockers were found to be associated with self-reported symptoms of anxiety. However, of these drugs neuropsychiatric side effects in relation to anxiety were only reported for antiarrhythmics and beta-blockers. Further studies investigating the association between antiarrhythmics, beta-blockers and anxiety are recommended. This study adds knowledge to the existing evidence of the associations between cardiac drug therapy and anxiety.

## Supplementary Information

**Additional file1:** **Table S1.** List of the included cardiac drug therapies. **Table S2.** List of the included psychotropic medication.

## Data Availability

The datasets generated and/or analysed during the current study are not publicly available due to participants’ right to anonymisation, but are available in an anonymised form, from the corresponding author on reasonable requests.

## Abbreviations

HADS: Hospital anxiety and depression scale
ICD: Implantable cardioverter-defibrillator
CRS: The Danish civil registration system
DMA: The Danish medicines agency
EMA: The European medicines agency
DNPR: The Danish national prescription registry
ATC: Anatomical therapeutic chemical classification code
BMI: Body mass index
SD: Standard deviations
P value: Probability value
OR: Odds ratio
CI: Confidence interval

## References

[1] Pogosova N, Kotseva K, De Bacquer D, von Känel R, De Smedt D, Bruthans J, Dolzhenko M (2017). Psychosocial risk factors in relation to other cardiovascular risk factors in coronary heart disease: results from the EUROASPIRE IV survey. A registry from the European society of cardiology. Euro J Prev Cardiol.

[2] Berg SK, Rasmussen TB, Thrysoee L, Lauberg A, Borregaard B, Christensen AV (2017). DenHeart: differences in physical and mental health across cardiac diagnoses at hospital discharge. J Psychosom Res.

[3] Hanssen TA, Nordrehaug JE, Eide GE, Bjelland I, Rokne B (2009). Anxiety and depression after acute myocardial infarction: an 18-month follow-up study with repeated measures and comparison with a reference population. Eur J Cardiovasc Prev Rehabil Off J Eur Soc Cardiol Work Groups Epidemiol Prev Card Rehabil Exerc Physiol.

[4] Celano CM, Millstein RA, Bedoya CA, Healy BC, Roest AM, Huffman JC (2015). Association between anxiety and mortality in patients with coronary artery disease: a meta-analysis. Am Heart J.

[5] Zigmond AS, Snaith RP (1983). The hospital anxiety and depression scale. Acta Psychiatr Scand.

[6] Celano CM, Daunis DJ, Lokko HN, Campbell KA, Huffman JC (2016). Anxiety disorders and cardiovascular disease. Curr Psychiatry Rep.

[7] Brennan C, Worrall-Davies A, McMillan D, Gilbody S, House A (2010). The hospital anxiety and depression scale: a diagnostic meta-analysis of case-finding ability. J Psychosom Res.

[8] Berg SK, Herning M, Thygesen LC, Cromhout PF, Wagner MK, Nielsen KM (2019). Do patients with ICD who report anxiety symptoms on hospital anxiety and depression scale suffer from anxiety?. J Psychosom Res.

[9] Zhang L, Bao Y, Tao S, Zhao Y, Liu M (2022). The association between cardiovascular drugs and depression/anxiety in patients with cardiovascular disease: a meta-analysis. Pharmacol Res.

[10] Huffman JC, Stern TA (2007). Neuropsychiatric consequences of cardiovascular medications. Dialog Clin Neurosci.

[11] Shah K, Parekh N, Clopton P, Anand I, Christenson R, Daniels L (2013). Improved survival in patients with diastolic heart failure discharged on beta-blocker and ace inhibitors. J Am Coll Cardiol.

[12] Bangalore S, Messerli FH, Kostis JB, Pepine CJ (2007). Cardiovascular protection using beta-blockers: a critical review of the evidence. J Am Coll Cardiol.

[13] Cojocariu SA, Maștaleru A, Sascău RA, Stătescu C, Mitu F, Leon-Constantin MM (2021). Neuropsychiatric consequences of lipophilic beta-blockers. Medicina.

[14] Martino E, Bartalena L, Bogazzi F, Braverman LE (2001). The effects of amiodarone on the thyroid. Endocr Rev.

[15] Thompson TS, Barksdale DJ, Sears SF, Mounsey JP, Pursell I, Gehi AK (2014). The effect of anxiety and depression on symptoms attributed to atrial fibrillation. PACE Pacing Clin Electrophysiol.

[16] Young-Xu Y, Chan A, Liao JK, Ravid S, Blatt CM (2003). Long-term statin use and psychological well-being in the elderly. J Am Coll Cardiol.

[17] Hoogwegt MT, Kupper N, Theuns DAMJ, Jordaens L, Pedersen SS (2012). Beta-blocker therapy is not associated with symptoms of depression and anxiety in patients receiving an implantable cardioverter-defibrillator. Europace.

[18] Berg SK, Svanholm J, Lauberg A, Borregaard B, Herning M, Mygind A (2014). Patient-reported outcomes at hospital discharge from Heart Centres, a national cross-sectional survey with a register-based follow-up: the DenHeart study protocol. BMJ Open.

[19] Pedersen CB (2011). The danish civil registration system. Scand J Public Health.

[20] Jensen VM, Rasmussen AW (2011). Danish education registers. Scand J Public Health.

[21] Lynge E, Sandegaard JL, Rebolj M (2011). The danish national patient register. Scand J Public Health.

[22] Tu JV, Austin PC, Walld R, Roos L, Agras J, McDonald KM (2001). Development and validation of the ontario acute myocardial infarction mortality prediction rules. J Am Coll Cardiol.

[23] Kildemoes HW, Sorensen HT, Hallas J (2011). The danish national prescription registry. Scand J Public Health.

[24] The Danish Medicines Agency [Internet]. 2019 [cited 2020 Sep 24]. Available from: https://laegemiddelstyrelsen.dk/en/sideeffects/side-effects-of-medicines/.

[25] The Danish Medicines Agency. Produc summaries [Internet]. [cited 2020 Nov 25]. Available from: http://www.produktresume.dk/AppBuilder/search.

[26] E. Commision, European Directorate-General E and I. A guideline on summary of product charateristics (SmPC) [Internet]. 2009. Available from: https://ec.europa.eu/health/sites/health/files/files/eudralex/vol-2/c/smpc_guideline_rev2_en.pdf.

[27] E. Commision, European Directorate-General E and I. A guideline on summary of product charateristics (SmPC). 2009.

[28] The Danish Medicines Agency. Produc summaries.

[29] Danish Cardiological Society. No Title [Internet]. [cited 2020 Oct 12]. Available from: https://nbv.cardio.dk/.

[30] Marinakis C. Drug side effects [Internet]. [cited 2020 Oct 10]. Available from: https://pro.medicin.dk/Specielleemner/Emner/500.

[31] Christensen AV, Dixon JK, Juel K, Ekholm O, Rasmussen TB, Borregaard B (2020). Psychometric properties of the Danish hospital anxiety and depression scale in patients with cardiac disease: results from the DenHeart survey. Health Qual Life Outcomes.

[32] Delong JM, Moser KD, Riegel B (2008). Impact of anxiety on cardiac disease. Cardiac Nursing: a companion to Braunwald’s heart disease.

[33] The Danish Medicines Agency. Information on product summaries [Internet]. [cited 2020 Nov 24]. Available from: https://laegemiddelstyrelsen.dk/da/bivirkninger/find-medicin/produktresumeer/.

[34] Molero Y, Cipriani A, Larsson H, Lichtenstein P, D’Onofrio BM, Fazel S (2020). Associations between statin use and suicidality, depression, anxiety, and seizures: a Swedish total-population cohort study. Lancet Psychiatry.

[35] Matsuo Y, Kasama S, Toyama T, Funada R, Takama N, Koitabashi N (2016). Comparative effects of long-acting and short-acting loop diuretics on cardiac sympathetic nerve activity in patients with chronic heart failure. Open Hear.

[36] Leutner M, Matzhold C, Kautzky A, Kaleta M, Thurner S, Klimek P, Kautzky-Willer A (2021). Major depressive disorder (MDD) and antidepressant medication are overrepresented in high-dose statin treatment. Front Med.

[37] De Hert M, Detraux J, Vancampfort D (2018). The intriguing relationship between coronary heart disease and mental disorders. Dialogues Clin Neurosci.

