# Supplementary material for: The association between cardiac drug therapy and anxiety among cardiac patients: results from the national DenHeart survey
Source: BMC Cardiovasc Disord. 2022 Jun 20;22:280. doi: 10.1186/s12872-022-02724-4 (PMC9210711; doi:10.1186/s12872-022-02724-4)
Supplement: Supplementary file 1 — Additional file1: Table S1. List of the included cardiac drug therapies. Table S2. List of the included psychotropic medication. [file 12872_2022_2724_MOESM1_ESM.pdf]

Supplementary material

**Supplementary Table 1:** List of the included cardiac drug therapies	
**Diuretics (C03)**	Hydrochlorthiazide, Metolazone, Bumetanide, Furosemide, Spironolactone and Eplerenone
**Lipid-lowering Agents (C10AA)**	Simvastatin, Pravastatin, Fluvastatin, Atorvastatin and Rosuvastatin
**Aspirins (B01AC06**)	Acetylsalicylic acid
**Anticoagulants (B01A)**	Phenprocoumon, Warfarin, Pradaxa, Eliquis, Xarelto and Lixiana
**Aldosterone antagonists (C03DA)**	Spironolacton and Eplerenon
**Nitrates (C01DA)**	Isorbide dinitrate
**Antiarrhythmics (C01B)**	Amiodaron, Dronedaron, Flecainid, Verapamil, Propafenon and Sotalol
**Digoxin (C01AA)**	Digoxin
**Angiotensin receptor antagonists (C09CA)**	Losartan, Irbesartan, Candesartancilexetil, Valsartan, Telmisartan and Olmesartanmedoxomil
**Angiotensin receptor antagonists (C09CA)**	Losartan, Irbesartan, Candesartancilexetil, Valsartan, Telmisartan and Olmesartanmedoxomil
**ACE-inhibitors (C09AA)**	Lisinopril, Captopril, Enalapril, Perindopril, Trandolapril and Ramipril
**Beta-blockers (C07)**	Atenolol, Bisoprolol, Metoprolol, Esmolol, Nebivolol, Landiolol, Propranolol and Sotalol

Supplementary Table 2: List of the included psychotropic medication

Psychotropic medication	ATC-code
Selective Serotonin Reuptake Inhibitors	N06AB
Tricyclic Antidepressants	N06AA
Serotonin–norepinephrine reuptake inhibitor	N06AX
Benzodiazepines	N05BA, N05CD, N03AE01, N05CF
Melatonin	N05CH
First generation antipsychotics and second-generation antipsychotics	N05A, N05AX08, N05AX12, N05AX13, N05AL05, N05AH02, N05AH03, N05AE04, N05AN01, N05AH05, N0AE03, N05AE04
